# 

**Published:** 1905-09

**Authors:** 


*THE BRISTOL MEDICO-CHIRURGICAL SOCIETY.*

CONTENTS.

| | PAGE |
|---|---|
| **The Relation of Medicine to the Natural Sciences.** By J. Michell Clarke, M.A., M.D. Cantab., F.R.C.P. | 193 |
| **The Surgical Aspect of Cholelithiasis.** By James Swain, M.S., M.D. Lond., F.R.C.S. | 203 |
| **Twin X-Ray Representation and the Reflecting Stereoscope.** By William Cotton, M.D. Edin. | 216 |
| **A Study of the Records of One Hundred and Fifty-five Cases of Operation for Appendicitis.** By Charles A. Morton, F.R.C.S. | 223 |
| **A Note on Congenital Dilatation of the Ureters with Hydronephrosis.** By J. M. Fortescue-Brickdale, M.A., M.D. Oxon. | 231 |
| **On Some Anomalous Cases of Locomotor Ataxy.** By F. H. Edgeworth, M.B. Cantab., D.Sc. Lond. | 234 |
| **Progress of the Medical Sciences:** | |
| **Medicine.** Newman Neild, M.B., B.Ch. Vict. | 239 |
| **Surgery.** T. Carwardine, M.B., M.S. Lond. | 244 |
| **Ophthalmology.** C. H. Walker, M.B., B.A. Cantab. | 249 |
| **Reviews of Books** | 254 |
| **Editorial Notes** | 270 |
| **Notes on Preparations for the Sick** | 278 |
| **The Library of the Bristol Medico-Chirurgical Society** | 281 |
| **Obituary Notices** | 284 |
| **Local Medical Notes** | 285 |

